# The Role of Probiotics and Their Postbiotic Metabolites in Post-COVID-19 Syndrome

**DOI:** 10.3390/molecules30204130

**Published:** 2025-10-20

**Authors:** Monika E. Jach, Ewa Sajnaga, Marharyta Bumbul, Anna Serefko, Kinga K. Borowicz, Hieronim Golczyk, Marek Kieliszek, Adrian Wiater

**Affiliations:** 1 Department of Molecular Biology, Medical Faculty, The John Paul II Catholic University of Lublin, Konstantynów Street 1I, 20-708 Lublin, Poland; h.golczyk@wp.pl; 2 Department of Biomedicine and Environmental Research, Medical Faculty, The John Paul II Catholic University of Lublin, Konstantynów Street 1J, 20-708 Lublin, Poland; esajnaga@kul.pl; 3 Medical Faculty, The John Paul II Catholic University of Lublin, Konstantynów Street 1I, 20-708 Lublin, Poland; marvettss46@gmail.com; 4 Department of Clinical Pharmacy and Pharmaceutical Care, Medical University of Lublin, Chodźki Street 1, 20-093 Lublin, Poland; anna.serefko@umlub.pl; 5 Independent Unit of Experimental Neuropathophysiology, Department of Toxicology, Medical University of Lublin, Jaczewskiego 8b, 20-090 Lublin, Poland; kingaborowicz@umlub.pl; 6 Department of Food Biotechnology and Microbiology, Institute of Food Sciences, Warsaw University of Life Sciences-SGGW, Nowoursynowska Street 159C, 02-776 Warsaw, Poland; marek-kieliszek@wp.pl; 7 Department of Industrial and Environmental Microbiology, Institute of Biological Sciences, Maria Curie-Skłodowska University, ul. Akademicka 19, 20-033 Lublin, Poland; adrian.wiater@mail.umcs.pl

**Keywords:** antiviral mechanisms, gut–brain axis, gut–lung axis, long COVID, microbiota, post-COVID, probiotics, postbiotics, SARS-CoV-2, post-acute COVID-19 syndrome, SCFAs

## Abstract

Post-COVID-19 syndrome, also known as long-COVID, is characterized by a wide spectrum of persistent symptoms involving multiple body organs and systems, including fatigue, gastrointestinal disorders, and neurocognitive dysfunction. Emerging evidence suggests that gut microbiota dysbiosis and disruption of the gut–brain axis play a central role in the pathophysiology of this condition. Probiotics and their metabolites (postbiotics) have gained increasing attention as potential therapeutic agents given their immunomodulatory, anti-inflammatory, and antiviral properties. In this review, we discuss the current understanding of the antiviral mechanisms of probiotics, including reinforcement of intestinal epithelial barrier function, direct virus inhibition, receptor competition, and immune system modulation. Special emphasis is placed on short-chain fatty acids (SCFAs), lactic acid, hydrogen peroxide, and bacteriocins as key factors that contribute to these effects. SCFAs appear to be essential postbiotic compounds during post-COVID recovery. We also highlight recent clinical trials involving specific probiotic species, such as *Lactiplantibacillus plantarum*, *Lacticaseibacillus rhamnosus*, and *Bifidobacterium longum*, and their potential role in alleviating long-term COVID symptoms. Although the current results are promising, further research is needed to clarify the most effective strains, dosages, and mechanisms of action in post-COVID therapeutic strategies.

## 1. Introduction

The COVID-19 (Coronavirus disease 2019) pandemic caused by Severe Acute Respiratory Syndrome Coronavirus 2 (SARS-CoV-2) has had a significant impact on global public health, healthcare, and social structures, with more than 259 million cases of the illness worldwide [1,2,3,4]. Hospitals experienced significant overcrowding, and healthcare personnel operated under extreme strain. The general population was compelled to undergo substantial lifestyle adjustments. Prolonged social isolation, sustained restrictions on mobility, and the obligatory use of face masks have had a measurable impact on both the psychological and physiological well-being of individuals. Many of patients who have survived the acute phase of SARS-CoV-2 infection experience long-term symptoms, collectively known as “post-acute COVID-19 syndrome” (PACS), “post-COVID-19 syndrome” (PCS), “post-COVID”, or “long-COVID”, which persist for weeks, months, or longer. Post-COVID can affect various physiological systems, causing fatigue, headache, shortness of breath, psycho-emotional disturbances, and gastrointestinal (GI) symptoms [5,6,7,8,9,10,11,12,13]. COVID-19 patients experience a significant change in the composition and function of their natural gut microbiota, causing dysbiosis, which has been shown to correlate with the severity of viral infection [14]. Specifically, reduction in beneficial taxa, such as *Faecalibacterium prausnitzii* and *Bifidobacterium* spp., and enrichment of potentially pathogenic bacteria, including *Enterococcus*, *Klebsiella*, and *Streptococcus*, have been reported [15,16,17,18,19]. Such dysbiosis may exacerbate systematic inflammation, compromise the integrity of the intestinal barrier, and modulate neuroimmunological signaling, all of which are implicated in post-COVID symptomatology [20].

Studies have indicated that intake of probiotics is effective in treating intestinal dysbiosis. Live microorganisms with probiotic properties and their health-promoting metabolites, known as postbiotics, positively affect the health of the host by restoring the balance of the microbiota and promoting immunity [21]. There is a growing body of evidence that some strains of probiotic bacteria may also exert antiviral, immunomodulatory, and barrier-enhancing effects [22]. In addition to probiotics, postbiotics, such as short-chain fatty acids (SCFAs), lactic acid, hydrogen peroxide (H_2_O_2_), and bacteriocins, are being investigated for their capacity to modulate host responses and support recovery. Mechanistic insights into how these compounds regulate cytokine production, influence tight junction (TJ) protein expression, and alter mucosal immunity are expanding rapidly [23,24,25,26]. Therefore, the use of probiotics and bioactive postbiotic metabolites may be a potential therapeutic option for improving patient health and reducing the severity or eliminating the symptoms of post-COVID.

This paper aims to highlight the connection between lungs, digestive and mental health, specific symptoms of post-COVID, and the severity of the disease. It also emphasizes the importance of the microbiota in COVID-19 and explores the potential benefits of probiotics and postbiotics in the prevention, management, and treatment of post-COVID. Emphasis is placed on molecular mechanisms, clinical trial outcomes in the pandemic period until mid-2025, and future directions in this emerging field. A particular focus is given to the potential of microbiota-targeted therapies to alleviate fatigue, GI symptoms, and neurocognitive dysfunction associated with post-COVID.

## 2. Methodology of the Review—Literature Search Strategy

The literature used in this review was collected over a period of two months using databases and platforms available through the library system of the John Paul II Catholic University of Lublin (KUL), including Scopus, PubMed, EBSCO, Google Scholar, De Gruyter, Springer Nature, Oxford University Press-Medicine Collection, SAGE Premier, Science, SpringerLink, Taylor & Francis, Wiley Online Library, and open repositories. The search covered publications from January 2020 to June 2025. The keywords were used individually and in combinations using Boolean operators and included: “probiotics,” “postbiotics,” “metabolites,” “randomized controlled trials,” “meta-analyses,” “post-acute COVID-19 syndrome,” “post-COVID,” “long COVID,” and “COVID-19.” The inclusion criteria focused on clinical trials (RCTs), meta-analyses, and mechanistic reviews on the gut microbiota and its modulation in post-COVID-19 recovery. Non-peer-reviewed articles, case reports, and publications not available in English were excluded.

## 3. COVID-19 Pandemic and Its Impact on the Natural Human Microbiota

### 3.1. Main Aspects of COVID-19 Incidence

The first cases of SARS-CoV-2 coronavirus (*Coronaviridae* family) infection, which causes acute respiratory failure, were reported in December 2019 in Wuhan, China. The virus has had a huge impact worldwide, spreading extremely rapidly in a large number of countries. As a result, on 11 March 2020, the World Health Organization (WHO) declared a pandemic of the disease dubbed COVID-19, which killed more than 6 million people between December 2019 and August 2022 [27,28].

Although significant advances in clinical research have provided a deeper understanding of SARS-CoV-2 infection, many countries are still facing new outbreaks. The virus that causes COVID-19, like other RNA viruses, has demonstrated a significant ability to evolve and evade the host immune response through genetic evolution, resulting in mutant forms with distinct characteristics. The process of mutations can lead to the emergence of variants that escape neutralization by the immune system [29,30,31]. The virus undergoes changes in its genome structure, such as depletion of CpG dinucleotides, which may help it evade the host’s antiviral defenses. These changes suggest adaptation towards a more homogeneous genome structure, facilitating evasion of the immune response [32]. The SARS-CoV-2 spike (S) protein, which is crucial for the virus to enter host cells, undergoes rapid mutations. These mutations can alter the antigenicity of the virus, making it more difficult for the immune system to recognize and neutralize the virus. For example, the D614G mutation in the S protein has been linked to increased transmissibility and immune evasion [33,34,35,36]. Coronavirus 2 continues to mutate, manifesting multiple SARS-CoV-2 variants, such as Alpha (B. 1.1.7), Beta (B. 1.351), Gamma (P. 1), Delta (B. 1.617.2), Omicron (B. 1.1.529) [37], and the Eris (EG.5) mutation derived from the XBB Omicron strain, which was detected in July 2023 and dominated for the next 12 months [38]. Different variants of concern (VOCs), e.g., Alpha, Delta, and Omicron, exhibit different replication and pathogenicity characteristics. For example, the Delta variant exhibits increased syncytium formation and severe cell damage compared to the Alpha and Omicron variants, which may contribute to its higher pathogenicity [39,40]. Moreover, Delta variants have developed mechanisms that suppress the host’s innate immune response, leading to delayed or weakened immune system intervention. This suppression can result in more severe symptoms and increased transmissibility [40].

Since July 2024, the fastest-spreading mutation was FLiRT—the SARS-CoV-2 variant that included strains KP.2 and JN.1, which also belong to the Omicron group [41]. As of December 2024, LP 1.8 was the dominant variant [42]. The newest variant of SARS-CoV-2, designated NB.1.8.1, was first identified in the US in late March and early April 2025. NB.1.8.1 has been recognized as a variant under monitoring (VUM) with increasing proportions globally, while LP.8.1 is starting to decline. Considering the available evidence, the additional public health risk posed by NB.1.8.1 is evaluated as low at the global level. Notwithstanding the simultaneous increase in cases and hospitalizations in some countries where NB.1.8.1 was widespread, the current data do not indicate that this variant causes more severe illness than the other variants [43]. By the end of May 2025, over 777 million people worldwide had been infected with COVID-19 [3], indicating that nearly 1/8 of all the people in the world had experienced this disease.

COVID-19 does not discriminate by age and can affect anyone. However, some studies [44,45] have shown that older individuals and those with chronic comorbidities are more susceptible to severe forms of the disease and its complications. The primary target of SARS-CoV-2 is the respiratory system. Upon entering through the respiratory tract, the virus initially attacks the lining of the upper airways, such as the nasal cavity and throat. It can then spread to the lower respiratory tract, causing inflammation and damage to the bronchi, lungs, and alveoli [46]. This can lead to the development of pneumonia and acute respiratory disorders, potentially resulting in acute respiratory failure. Furthermore, some patients experience an overactive immune response, which may lead to a severe form of pneumonia known as a cytokine storm, further worsening the patient’s condition [47]. Extrapulmonary symptoms may also occur during the course of the disease, causing changes in the cardiovascular, genitourinary, digestive, and central nervous systems (CNS) [48].

Most infected individuals have only mild or no symptoms. The majority of patients with mild COVID-19 recover within a week or two without major complications. This scenario is particularly typical of young and healthy individuals without coexisting medical problems. In fact, such individuals or those who have undergone a more complex form of the disease may develop post-COVID. The symptoms of this syndrome can last several weeks, months, or even longer after recovery from the underlying COVID-19 disease [49,50]. In 2021, Kamal et al. [51] conducted a study in which people who had recovered from SARS-CoV-2 infection were given questionnaires to collect data on post-COVID symptoms. They found that only 10.8% of the study participants had no symptoms of post-COVID. The other participants reported such conditions as fatigue and weakness, mood changes (i.e., depression and anxiety), muscle and joint pain, memory and concentration problems, and shortness of breath. Some patients had experienced more critical conditions, such as stroke, pulmonary fibrosis, and kidney failure. Other researchers reported that patients also complained of headaches, impaired memory and concentration called “COVID fog” or brain fog, sleep problems such as difficulty falling asleep, waking up during the night, sleep that is too short and unrestorative, chest pain, problems with taste and/or smell, and GI disorders [50]. It is estimated that more than 65 million people suffer from post-COVID [52]. As new COVID-19 cases are still being reported [3], this number can be expected to continue to increase.

### 3.2. Gut Microbiota in COVID-19

#### 3.2.1. Role of Microbiota in Maintaining Health

Since birth, the human body has maintained a symbiotic relationship with the diverse community of microorganisms that are essential for host survival and play a crucial role in host metabolism, immunity, and homeostasis. This complex assemblage, primarily composed of bacteria, archaea, fungi, virome, and protozoa, is referred to as the “microbiota” [53]. In turn, the term “microbiome” encompasses the collective genetic material, physicochemical characteristics, and metabolic interactions of these microorganisms, which contribute to the formation of distinct ecological niches within the host environment [54]. These microbial communities colonize various anatomical sites, adapting to local conditions. To date, microorganisms have been identified on mucosal surfaces, the skin, the GI tract, respiratory and urinary tracts, mammary glands, the vagina, and even the placenta [55,56].

Studies have demonstrated that the microbiota of each healthy individual constitutes a unique and non-replicable ecosystem within the body [57,58]. Its composition evolves throughout the human life cycle [59], with particularly dynamic shifts occurring during the early years of life and in response to disease states [59,60]. A multitude of factors shape the microbiota’s structure and diversity. Notably, the mode of delivery, type of infant feeding, and maternal lifestyle significantly influence the initial microbial colonization of the newborn [59]. Infants delivered via cesarean section tend to exhibit a reduced microbial diversity, with a composition resembling the skin microbiota, characterized by the predominance of such genera as *Staphylococcus*, *Corynebacterium*, and *Propionibacterium*. In contrast, vaginally delivered infants display a more diverse microbiota, closely resembling that of the maternal intestinal and vaginal microbiota, and are enriched in *Lactobacillus* sensu lato (s.l.), *Prevotella*, and *Sneathia* [61]. Lifestyle factors and environmental exposures continue to modulate the microbiota composition throughout life. For instance, comparative studies have identified differences in the microbiome profiles of rural versus urban populations, potentially associated with occupational activities such as office versus field work [62]. Antibiotic administration represents another major factor influencing the microbiota composition. While the gut microbiota of adults is generally resilient to short-term antibiotic exposure, repeated or prolonged antibiotic use may disrupt microbial balance, thereby promoting the overgrowth of opportunistic organisms [59].

The microbiota lives in large numbers in the human digestive tract. The species diversity and the numbers of microbes vary considerably in different parts of the GI tract. The main oral microbiota includes six types: *Bacillota*, *Pseudomonadota*, *Bacteroidota*, *Actinomycetota*, *Fusobacteriota*, and *Spirochaetota*, accounting for 96% of all bacteria [63]. The esophageal microbiota is mainly dominated by bacteria from the genera *Streptococcus*, *Prevotella*, and *Veillonella* [64]. The stomach is the primary organ of the digestive system. The acidic environment of this organ kills many bacteria and viruses; nevertheless, it is inhabited by a wide variety of microorganisms. Bacteria from the genera *Enterococcus*, *Streptococcus*, *Staphylococcus*, and *Stomatococcus* are commonly found in the stomach [65].

The gut microbial community, is even more diverse and numerous, particularly in the large intestine. Currently, the GI microbiota is considered a very significant ally of the body, as it is assumed to affect not only the function of the intestine but also the activity of other organs, including the brain via the gut–brain axis [66]. Research results indicate that the main types of microorganisms inhabiting the gut are *Bacillota* (*Clostridium*, *Lactobacillus* s.l., *Ruminococcus*, and *Eubacterium* genera), *Bacteroidota* (*Bacteroides* and *Prevotella* genera), *Pseudomonadota* (*Enterobacter* spp.), and *Actinomycetota (Bifidobacterium* and *Collinsella* genera) [57]. The gut microbiota is highly diverse, allowing its constituents to perform various functions. Gut microbes are capable of metabolizing proteins and bile acids, biosynthesizing certain hormones, amino acids, and vitamins (e.g., C, K, B12, and folic acid), and fermenting indigestible carbohydrates to produce SCFAs [67]. The microbiota can protect the gut from pathogens by producing antimicrobial substances, such as bacteriocins [22,53,67]. The GI microbiota plays a key role in modulating the human immune system. Approximately 80% of immunocompetent cells are located in the gut mucosa, making it the body’s largest immune organ. Through constant antigenic stimulation, the microbiota supports the development of both local and systemic immunity [53].

Some sources indicate that the gut bacterial ecosystem has a direct connection to the respiratory system through an organ interaction described as the “gut-lung axis” or “gut-lung-brain axis” [68]. Such a relationship is believed to occur in both ways, meaning that microbial metabolites and endotoxins present in the lungs can affect the gut microbiota composition [69]. Such an interaction between the gut and lungs has been recorded; however, the mechanisms of their mutual influence have not been fully explored and are at an early stage of research. These findings suggest that changes in the composition of the gut microbiota are related to increased susceptibility to respiratory diseases as well as changes in the immune response and lung homeostasis [70]. Considering the link between respiratory organs and the gut microbial community, it is possible to speculate that the novel coronavirus may also affect the composition of the gut microbiota.

#### 3.2.2. Microbiota in the Course of SARS-CoV-2 Infection

It has been suggested that there is a direct link between the state of the GI microbiota and SARS-CoV-2. Individuals affected by changes in the composition of the GI microbiota, observed as intestinal dysbiosis, may be predisposed to develop a more severe form caused by SARS-CoV-2 infection [71,72]. Importantly, dysbiosis manifests as a disturbance in not only the composition but also the function of microorganisms [73]. The condition is becoming increasingly common among humans, as it can be caused by a number of factors: an inappropriate diet rich in sugar and low in fiber, lifestyle changes (e.g., changes in physical activity levels), increased stress, various health conditions and infections, and the use of antibiotics. The propensity to develop a more severe form of COVID-19 is related to the following factors [74]: (1) the prevalence of opportunistic microorganisms in the gut, which increases the likelihood of complications; (2) a reduced level of beneficial bacteria with immunomodulatory effects; (3) an enhanced level of initial inflammation that increases the risk of immunopathology; (4) the development of “leaky gut syndrome” in the presence of a viral infection in the intestine. The results of a study conducted by Zuo et al. [75] showed that the GI microbiome of healthy individuals differed significantly from that of patients affected by COVID-19. These modifications in the microbiome were observed even in patients who eventually tested negative for SARS-CoV-2. This study showed a change in the fecal microbiome during SARS-CoV-2 infection and its correlation with disease severity. It was found that even patients with confirmed COVID-19 who had not previously received antibiotic therapy had an increased number of opportunistic bacteria, including *Clostridium hathewayi*, *Bacteroides nordii*, and *Actinomyces viscosus*, compared with healthy individuals. It was also observed that the presence of *Clostridium ramosum* and *C. hathewayi* indicated a more complex clinical presentation of COVID-19. At the same time, the anti-inflammatory bacterium *Faecalibacterium prausnitzii* and some representatives of the *Bacteroidota* phylum (e.g., *Bacteroides dorei*, *Bacteroides thetaiotaomicron*, *Bacteroides massiliensis*, and *Bacteroides ovatus*) were inversely correlated with disease severity. Moreover, COVID-19 patients exhibited a reduction in beneficial symbionts, such as *Roseburia* and *Lachnospiraceae*, and an increase in opportunistic pathogens like *Enterococcus* and *Proteobacteria*. This shift was associated with decreased alpha diversity, especially during the acute phase of the disease [19,76].

Rafiqul Islam et al. [77] examined the gut and oral microbiome of patients with SARS-CoV-2 infection and its association with the severity of COVID-19. The analysis, based on 16S ribosomal RNA gene sequencing, was conducted with 22 subjects with confirmed SARS-CoV-2 infection and 15 healthy individuals. According to the results, the diversity of the oral and GI microbiome of the COVID-19 patients differed significantly from that of the healthy individuals. An increased abundance of both pathogenic and commensal bacteria was observed in patients with COVID-19. Intestinal and oral samples from the COVID-19 patients had a higher relative abundance of bacterial genera *Bacteroides*, *Escherichia*, *Shigella*, *Enterococcus*, *Bifidobacterium*, *Megamonas*, *Streptococcus*, *Rothia*, *Klebsiella*, and *Veillonella*. Different levels of *Bacteroides*, *Prevotella*, *Escherichia*, *Shigella*, *Enterococcus*, *Bifidobacterium*, *Megamonas*, and *Actinomyces* were identified in the intestines of patients with COVID-19. This research team also noted the likely association of the microbiota with various complications in COVID-19 patients, including diarrhea. Patients with diarrhea had higher levels of GI pathogens, such as *Escherichia* and *Shigella*, than the controls. Interestingly, various species of *Prevotella* and *Veillonella* were over-represented in the COVID-19 patient populations. Previous studies reported an association between *Prevotella* and systematic infections, including low-grade systematic inflammation. *Prevotella* is thought to produce proteins that facilitate SARS-CoV-2 infection and increase the clinical severity of COVID-19 [78]. The increased abundance of *Prevotella* and *Veillonella* on the tongue of elderly and frail patients has also been linked to an increased risk of death from pneumonia [79,80].

In addition, Bucci et al. [81] demonstrated the feasibility of determining the form of COVID-19 disease by analyzing the composition of the stool and oral microbiome with an accuracy level exceeding 80%. This analysis method proved to be more accurate than using clinical biomarkers and information about comorbidities. Patients with severe disease exhibited an increase in *Enterococcus faecalis* and *Porphyromonas endodontalis*, whereas those with moderate severity were characterized by an increase in *Bacteroides caccae*, *Bacteroides fragilis*, and *Clostridium clostridioforme* in the stool and *Muribaculum intestinale* in the oral cavity.

Cui et al. [82] characterized the oral and intestinal microbiome and serum metabolite in 35 patients with confirmed SARS-CoV-2 one year after recovery. The microbiota of the one-year convalescents was restored but did not return to normal completely. During the recovery process, the microbial diversity gradually increased. Microorganisms producing butyric acid (the main SCFAs) and *Bifidobacterium* gradually increased, whereas the number of microorganisms producing lipopolysaccharides (LPS) gradually decreased. In addition, sphingosine-1-phosphate (SIP) levels, whose lower profiles are closely related to the inflammatory storm of COVID-19, increased significantly during the recovery process, aiding in the restoration of endothelial function and reduction in inflammation [82,83,84,85]. Furthermore, predictive models developed based on the microbiome and metabolites in patients at the time of hospital discharge achieved high accuracy in predicting neutralizing antibody levels after one year [82].

Generally, intestinal microbiota dysbiosis in COVID-19 was linked to elevated pro-inflammatory markers and a dysregulated immune response as well as severity and long-term symptoms [86,87]. Pro-inflammatory factors associated with COVID-19 dysbiosis may contribute to the hyper-inflammatory immune response seen in severe cases [88]. Functional pathways in the GI microbiota, such as L-tryptophan biosynthesis, were disrupted in severe COVID-19 cases [89]. Additionally, decreased levels of SCFAs like acetate, propionate, and butyrate exacerbate inflammation and oxidative stress, perpetuating the disease [1]. Moreover, severe cases showed more pronounced intestinal microbiota alterations compared to mild cases. Specific microbial profiles may predict disease severity [76,89]. In turn, mild cases of coronavirus disease 2019 did not seem to affect the gut microbiome of patients with ulcerative colitis (UC), although *Verrucomicrobiota* bacteria were underrepresented in patients who tested positive for COVID-19 [90].

### 3.3. Post-COVID: Long-Term Consequences of SARS-CoV-2 Infection

**Figure 1:**
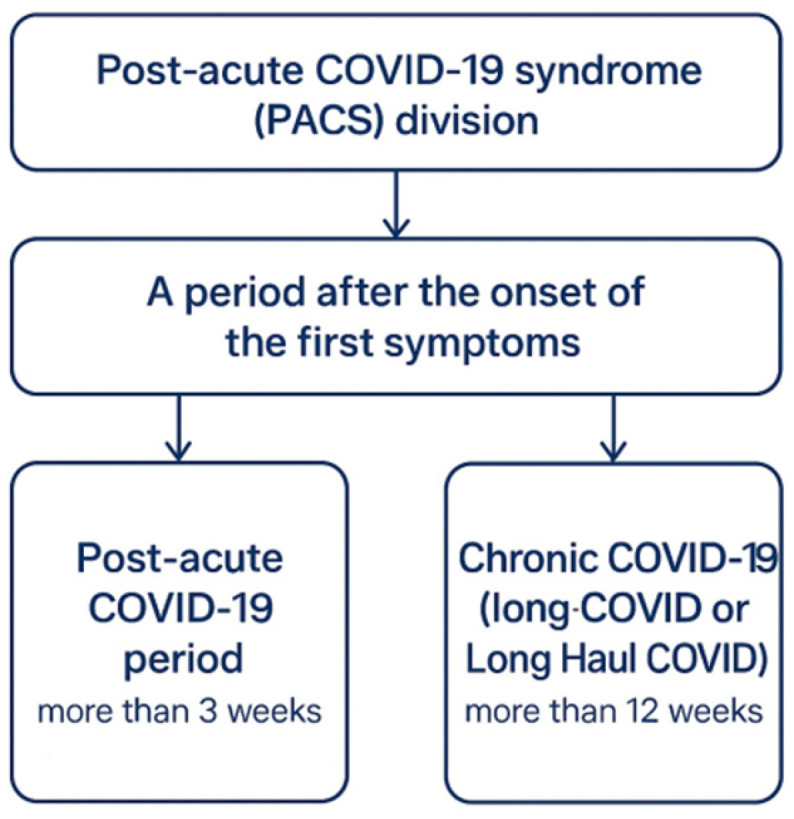
Division of post-acute COVID-19 syndrome (PACS) [68,93].

There are numerous publications from many regions of the world indicating that a certain proportion of patients who have had COVID-19 continue to experience various health problems [49,50,51,52,91]. Post-COVID has been observed in at least 10% of patients recovering at home and possibly in up to 50% and even 70% of hospitalized patients [92]. Post-COVID is observed in all levels of disease severity, from asymptomatic SARS-CoV-2 infection to critically ill patients with full-blown COVID-19. Post-COVID is divided into two basic periods (Figure 1).

Post-COVID symptoms may manifest even two months or more after the initial SARS-CoV-2 infection and last for more than 3 weeks, months, or years [68,94]. Some authors have suggested dividing patients with persistent symptoms into three categories [95]:

1. Those who had acute respiratory distress syndrome and were initially hospitalized but now have long-term breathing problems;
2. Those who did not require initial hospitalization but now show signs of damage to organs and various systems, such as the respiratory, cardiovascular, or nervous systems;
3. Those who have not been hospitalized but still have persistent symptoms, often accompanied by fatigue without obvious signs of respiratory damage.

Post-COVID can simultaneously affect multiple body organs and systems, resulting in cardiovascular, endocrine, respiratory, cognitive, musculoskeletal, and GI disorders. More than 200 different symptoms have been associated with post-COVID, the most commonly reported of which are malaise, shortness of breath, fatigue, brain fog (COVID fog), autonomic dysfunction, headache, persistent loss of smell and/or taste, cough (chronic coughing), sleep problems, anxiety, depression, low-grade fever, sore throat, palpitations, dizziness, muscle weakness, muscle and joint pain, diarrhea, abdominal pain and nausea, and reduced exercise capacity [92,96,97,98]. Systematic reviews [99,100,101] that aimed to analyze the long-term effects of COVID-19 calculated the frequencies of the most commonly experienced post-COVID symptoms shown in Table 1.

Moreover, a meta-analysis carried out by Lopez-Leon et al. [101] showed that 80% of people who underwent SARS-CoV-2 infection had at least one of the entire lists of symptoms of post-COVID. Some patients also complained of GI distress, including diarrhea, loss of appetite, nausea, and vomiting. These symptoms may be due to the entry of SARS-CoV-2 into host cells via angiotensin-converting enzyme 2 (ACE2) receptors found in intestinal epithelial cells, which can interfere with the normal functioning of this organ [103].

#### 3.3.1. Post-COVID Patient-Specific Factors

The risk factors (Table 2) that can enhance post-COVID development include older age, female sex, pre-existing health conditions, and possibly genetic factors [6,7,104]. Advanced age is consistently identified as a significant risk factor for severe COVID-19 and post-COVID complications. Patients over 55 years of age, especially those with comorbidities, are at higher risk for severe COVID-19 and long-term complications [105,106,107,108]. Older age is also associated with a higher prevalence of post-COVID symptoms, such as fatigue, shortness of breath, and cognitive impairment [108,109]. In turn, younger people, although they usually experience a milder course of acute COVID-19, may still suffer from serious post-COVID symptoms, including mental health problems such as anxiety and depression [110,111].

Evidence suggests that, although women may experience less severe acute symptoms of COVID-19, they are more susceptible to long-term health problems, including persistent post-COVID and mental health issues. Socioeconomic factors further exacerbate these effects, highlighting the need for gender-specific health policies and support systems to address the unique challenges that women face post-COVID [115,116,118,119,120,121].

Comorbidities are another risk factor for COVID-19 and post-COVID. Diabetes and hypertension are often associated with more severe COVID-19 and a higher risk of long-term complications [105,107,108]. Patients with diabetes and hypertension are more likely to experience persistent symptoms, such as fatigue and breathing difficulties [108]. Severe obesity [112,113], chronic kidney disease, cardiovascular diseases, and neurodegenerative conditions [107,108] are also linked to worse COVID-19 outcomes and prolonged post-COVID symptoms. Pre-existing mental health disorders can exacerbate post-COVID symptoms and are associated with worse outcomes, including higher rates of depression and anxiety [112,114].

Elevated levels of inflammatory cytokines, i.e., IL-6, are associated with post-COVID, which includes such symptoms as loss of appetite and weight loss [106]. This suggests that the initial inflammatory profile may influence the post-COVID condition. Although the specific research on core microbiome profiles is limited, the overall impact of immune response and inflammation on post-COVID outcomes suggests that microbiota health may be an important factor [105,106].

Moreover, genetic factors, including congenital immune disorders and age-related inflammation, may play a significant role in determining health outcomes after COVID-19, influencing both the severity of the initial infection and the long-term consequences experienced by individuals [122]. Different genetic variants may contribute to post-COVID problems. For example, variants of genes controlling the components of the immune system, like IL-2 gene polymorphism, influence the markers of systemic inflammation and vascular regulation in post-COVID patients. Specific polymorphisms may lead to differences in inflammation and other molecular changes, potentially affecting long-term health outcomes [117]. Genetic instability, which is influenced by factors related to physical and mental health, may contribute to the diverse clinical symptoms of post-COVID. Gender-related differences in genetic instability have been observed, indicating a complex interaction between genetic and environmental factors [123]. Identification of genes and pathways may help explain the internal risk factors associated with severe cases. Notably, genetic variants interact with non-genetic risk factors, such as gender, cardiometabolic health, and socioeconomic status to influence the severity of COVID-19 and post-COVID. For example, the rs2268616 variant in the placental growth factor (PGF) gene has a stronger effect in men and people with type 2 diabetes [124].

#### 3.3.2. Mechanistic Insights into the Role of Microbiota in Post-COVID

The underlying mechanisms of post-COVID are multifactorial, involving chronic immune dysregulation, persistent viral particles, autonomic nervous system dysfunction, and mitochondrial impairment [104,125,126]. Post-COVID patients continue to show an altered gut microbiota composition, with specific signatures associated with decreased respiratory function up to 12 months following the acute disease [19]. Persistent dysbiosis may increase susceptibility to long-term complications [127]. The dysbiotic microbiota in patients with post-COVID is characterized by a decrease in beneficial bacteria that produce SCFAs (such as *Faecalibacterium*, *Ruminococcus*, *Dorea*, and *Bifidobacterium*), along with an increase in opportunistic bacteria (*Corynebacterium*, *Streptococcus*, *Enterococcus*). This dysbiosis, or microbial imbalance, worsens the clinical course of COVID-19 and is associated with post-COVID, a condition that affects a significant proportion of survivors [98]. Notably, the GI microbiota composition in post-COVID patients correlates with elevated levels of immune and inflammatory markers. Increased C-reactive protein (CRP), interleukin (IL-6), and tumor necrosis factor-alpha (TNF-α) levels have been associated with the presence of pathogenic bacterial taxa, supporting the hypothesis of gut-mediated systemic inflammation [15,128,129].

##### Gut-Immune Axis and Inflammatory Regulation

The gut-immune axis is a key communication pathway between the GI microbiota and the immune system, influencing various aspects of human health and disease. Hence, the GI microbiota has a huge impact on host’s immune homeostasis, and its disruption can cause systemic inflammation [130,131]. In post-COVID patients, one of the characteristic features is the persistent activation of the immune system, and microbial dysbiosis may be one of the key underlying factors. Specific bacterial strains have been shown to modulate cytokine production and immune cell function. For example, such SCFAs as butyrate and propionate can regulate Treg cell differentiation, inhibit nuclear factor kappa B (NF-κB) activation, and lower IL-6 levels [132].

The latest data indicate that COVID-19 survivors with an altered GI microbiota profile showed elevated levels of IL-17, TNF-α, and IL-1β, suggesting persistent mucosal and systemic inflammation [133,134,135]. In addition, reduced abundance of *Faecalibacterium prausnitzii*, a key anti-inflammatory commensal, was associated with more severe fatigue and gastrointestinal symptoms. The interaction between the gut and the immune system remains a major mechanism in the pathogenesis of post-COVID [15,19,133,136].

Dietary habits, including fiber and fermented food intake, also play a key role in shaping the inflammatory response. People with healthier baseline diets tend to recover more quickly, likely due to higher numbers of SCFA-producing bacteria and more resilient gut ecosystems [131,137,138].

In addition, GI disorders, such as irritable bowel syndrome (IBS), inflammatory bowel disease (IBD), and metabolic syndrome, have been observed following SARS-CoV-2 infection up to 8 months. The virus binds to ACE2 receptors in the GI tract, causing damage to the intestinal barrier and stimulating the production of pro-inflammatory cytokines. Thus, these conditions may result from prolonged activation of mucosal immunity, microbial translocation, and epithelial barrier dysfunction [139,140,141,142]. Changes in the GI microbiota may also contribute to the development of metabolic syndrome through mechanisms related to damage to the gut-blood barrier, cytokine storm, and intestinal vascular thrombosis [143]. However, the interaction between viral remnants, altered microbiota, and immune signaling requires further investigation.

##### Gut–Brain Axis and Neuropsychological Symptoms

The gut–brain axis, a bidirectional communication system between the GI tract and the CNS, plays a fundamental role in regulating mood, cognitive function, and stress response [66]. Neurocognitive symptoms, such as brain fog, memory impairment, and sleep disturbances, are commonly reported in post-COVID and may be related to dysregulation of the gut–brain axis. Iqbal et al. [144] proposed two mechanisms in the development of dysfunction in the gut–brain axis. The first mechanism is associated with defective ACE2 receptor function caused by SARS-CoV-2 virus infection, which leads to impaired activation of the mTOR pathway, decreased secretion of antimicrobial peptides (AMPs), and dysbiotic changes in the gut. Secondly, enteroendocrine cell function, intestinal permeability, and vagus nerve signaling are also disrupted, further contributing to gut–brain axis dysfunction. These disturbances are often accompanied by reduced levels of beneficial metabolites (i.e., SCFAs) and increased translocation of pathogens or pathogen products (i.e., LPS) into the systemic bloodstream circulation, which promotes the release of pro-inflammatory cytokines. Hence, neuroinflammation may also arise due to the translocation of pro-inflammatory mediators and pathogen products across the blood–brain barrier, exacerbated by GI dysbiosis [145,146]. Moreover, an altered hypothalamic–pituitary–adrenal (HPA) axis is associated with decreased levels of neurotransmitters and reduced vagus nerve stimulation, which may also lead to neuroinflammation and dysregulation of serum cortisol levels [144]. Changes in L-tryptophan metabolism, including shifts toward the kynurenine pathway, can affect serotonin synthesis and are dependent on the composition of the bacterial microbiota [147,148,149]. Moreover, SCFAs may cross the blood–brain barrier modulating microglial activity and exhibit anti-inflammatory and neuroprotective effects, while endotoxins derived from gut pathogens can induce neuroinflammation [150,151,152].

Alterations in microbial metabolites such as SCFAs, neurotransmitter precursors (e.g., L-tryptophan), and bile acids may disrupt the gut–brain axis and contribute to systemic inflammation and neuroinflammation, manifesting as fatigue, brain fog, and mood disturbances [153,154,155]. Therefore, the GI microbiota is closely linked to both the immunological and neuronal aspects of post-COVID pathogenesis. Understanding these mechanisms is crucial for identifying new interventions targeting the microbiota.

#### 3.3.3. Cardiovascular and Muscle Problems in Post-COVID

Cardiovascular abnormalities and disorders, which can have serious health consequences, have also been observed in patients with post-COVID. Cardiovascular symptoms during post-COVID include arrhythmia, myocarditis, pericarditis, coronary artery disease, and heart failure as periodic complications and chest pain, dyspnea, and a rapid heart rate [97,156]. Moreover, since the COVID-19 pandemic, the incidence of postural orthostatic tachycardia syndrome (POTS) in post-COVID patients has risen rapidly, adding an estimated 6–7 million new cases in the U.S. Before the pandemic, POTS affected from 0.5% to 1% of the U.S. population. POTS is a chronic, debilitating condition characterized by an excessive increase in the heart rate upon orthostatic challenge [157]. Such complications show that there is a need for long-term monitoring of patients with ongoing COVID-19 and in the post-acute period and for development of effective methods to prevent and treat similar symptoms.

SARS-CoV-2 can affect skeletal muscles, causing muscle problems that persist after recovery. Such disorders are primarily caused by persistent muscle dysfunction, which begins in the acute phase of infection and is characterized by reduced muscle protein synthesis due to hyper-inflammation, hypoxemia, and muscle cell infection. The main consequence of impaired muscle function is a reduction in the quality and volume of muscle mass, reduced capillarization, and mitochondrial dysfunction, which manifests as a feeling of muscle weakness and leads to limitations in daily activities and a deterioration in the quality of life [158].

## 4. Probiotics Versus COVID-19

### 4.1. Probiotics as a Way to Rebuild a Healthy Microbiota

**Figure 2:**
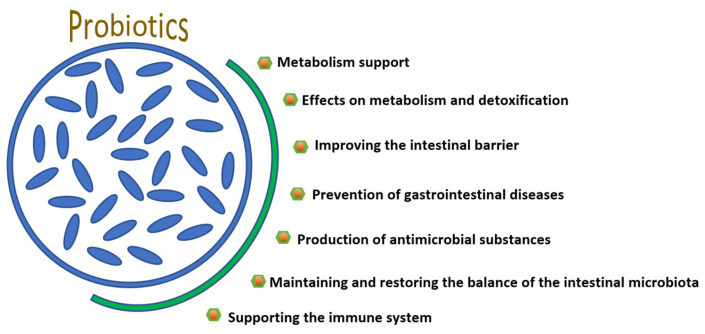
Impact of probiotics on the human body [160,161].

Probiotics are live microorganisms that, when consumed in sufficient quantities, have a beneficial effect on the host body by improving health and contributing to the maintenance of normal microorganisms (Figure 2). This definition was formulated in 2001 at a meeting of the Food and Agriculture Organization (FAO) and the WHO, and has since become the basis of scientific research [159].

The ideal probiotic for humans should, first and foremost, be of human origin and provide guaranteed safety, including the absence of pathogenicity, infectivity, deleterious metabolic activities, and excessive immune responses [162,163]. Furthermore, probiotics should not carry transferable antibiotic resistance genes [164]. In addition, it is essential for probiotics to have high GI viability, with the acidic effects of gastric juice, digestive enzymes, and bile salts. A key feature of probiotics is their antagonistic activity against various pathogens. Probiotic preparations should retain their activity and effectiveness after treatment with numerous industrial processing methods [160].

Probiotic bacteria can reside temporarily or permanently in the human GI tract, which is especially important when the normal microbiota is disrupted. Under such circumstances, exogenous strains of probiotic bacteria temporarily colonizing the GI tract can have a stabilizing effect on the composition and balance of the body’s microbiota, thereby restoring the key physiological function of the commensal microbiota [161].

Probiotics exhibit various mechanisms of action (Table 3). As mentioned earlier, probiotics primarily contribute to the restoration of normal GI microbiota in adults and children. While competing with pathogens in the gut for space and nutrients, they produce various antimicrobial substances, such as bacteriocins or organic acids, which inhibit the growth and multiplication of pathogens, thereby reducing the risk of infection. In addition, probiotics can modulate enzymatic activity, participating in the metabolism of various toxic substances and producing volatile fatty acids necessary to maintain energy balance. One of the important mechanisms of action of probiotics is their ability to increase intestinal cell adhesion, produce mucin (mucus), and regulate the activity of the immune system and intestinal lymphoid tissue [160].

Currently, gut microbiota rebalancing is considered clinically essential. Studies have shown that correcting microbiota imbalances can help improve well-being and treat various diseases, especially digestive and mental disorders, including depression [66]. There are many types of probiotic bacteria with beneficial properties. Lactic acid bacteria (LAB) (e.g., *Lactobacillus* s.l., *Lactococcus*, *Enterococcus*, and *Streptococcus*), *Bifidobacterium*, and *Bacillus* species as well as *Saccharomyces boulardii* yeasts are considered to be the best studied [165,166]. Probiotics are recommended for GI diseases because they contribute to positive health effects by lowering cholesterol levels, preventing diarrhea and intestinal discomfort, reducing inflammation, and inhibiting colon cancer and allergic reactions [167]. It should be emphasized that GI dysbiosis is transient and can be modulated through interventions, including probiotics and dietary supplements [144].

**Figure 3:**
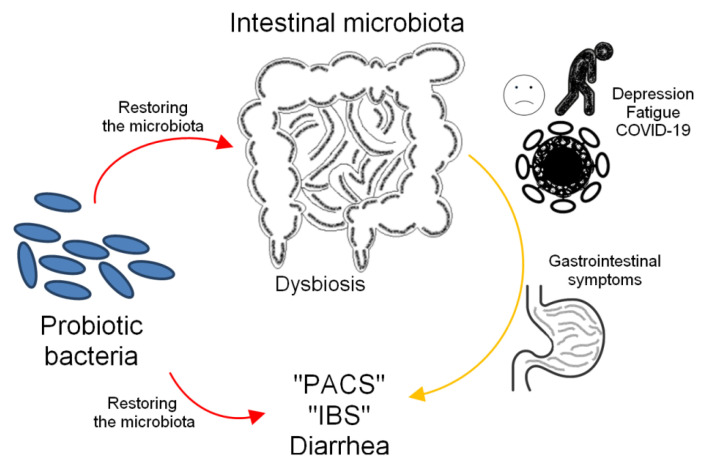
Role of probiotics in rebuilding intestinal microbiota, modulating immunity, and alleviating symptoms of fatigue, depression, PACS (post-acute COVID-19 syndrome), and IBS (Irritable Bowel Syndrome) [27,167,168,169,170,171].

Probiotics are commonly recommended for intestinal microbial dysbiosis (or dysbacteriosis) (Figure 3). According to some studies, pathological intestinal conditions, such as Irritable Bowel Syndrome (IBS) and Inflammatory Bowel Disease (IBD), are associated with intestinal dysbiosis [168]. IBD is the common name for two chronic diseases, UC and Crohn’s disease (CD), whose etiology is still not fully understood. Many studies have investigated the effects of probiotics on IBS. Both negative and positive results have been observed; however, the use of probiotics appears to be a promising therapeutic strategy for alleviating the symptoms of this disease. A critical review [169] noted that administration of *S. boulardii* to patients with CD and the use of *Escherichia coli* Nissle1917, *Bifidobacterium breve*, *Bifidobacterium bifidum*, and *Lactobacillus acidophilus* strains in UC promoted the maintenance of remission. This study also highlights that the use a mixture of several probiotic strains may be more effective than using each strain alone.

A fairly common consequence of antibiotic use is diarrhea, known as antibiotic-associated diarrhea (AAD), or post-antibiotic diarrhea, which affects up to one-third of patients. AAD can manifest with symptoms ranging from mild diarrhea to severe intestinal infections. The symptom complex is characterized by frequent watery stools, bloating, abdominal pain, nausea, and vomiting. It has been suggested that probiotics may be effective in preventing AAD. A systematic review and meta-analysis [171] found a 51% reduction in the risk of AAD induced by probiotics, with no apparent increase in the risk of side effects. In this meta-analysis, *Lacticaseibacillus rhamnosus* GG and *S. boulardii* were found to be the most effective probiotic strains.

### 4.2. Antiviral Effects of Probiotics

Currently, viral infections remain a serious public health challenge. By affecting the respiratory, and digestive systems, skin, and other tissues, viruses can lead to serious health consequences, including death. The continuing threat of pandemic viruses, such as the new coronaviruses, underscores the need for regular monitoring and control of such infections. The emergence of drug-resistant strains of viruses complicates this situation and undermines the effectiveness of traditional treatments. Recently, probiotics, known for their positive effects on the human GI tract and health in general, have become an increasingly interesting topic for scientists given their potential to combat various viral infections. Specialists are studying the mechanisms of action of these preparations against viruses and identifying specific probiotic strains exhibiting antiviral activity [172,173,174].

#### 4.2.1. Molecular Mechanisms of Microbiota Modulation in Post-COVID

**Figure 4:**
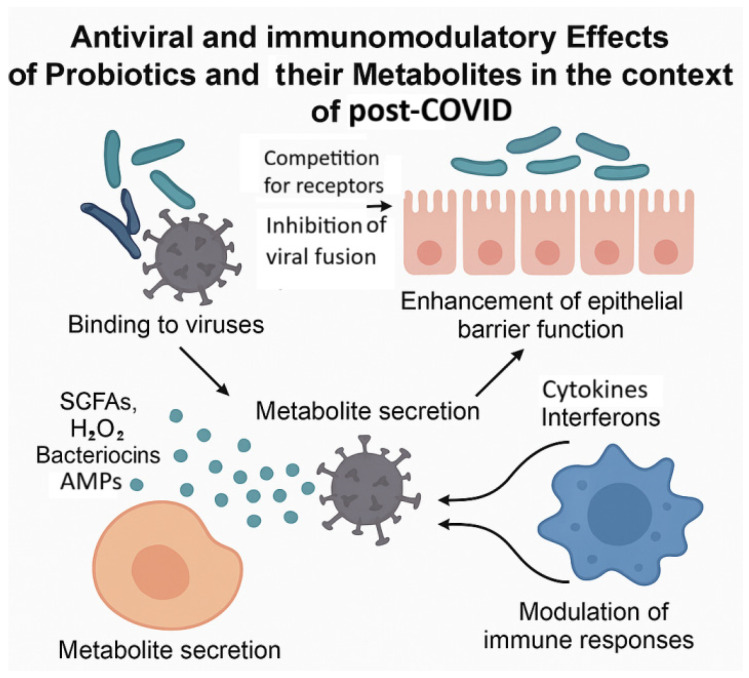
Antiviral and immunomodulatory effects of probiotics and postbiotics on post-COVID [172,173,179,180,181,182,183].

Microbiological modulation of host physiology in post-COVID involves a network of molecular mechanisms that affect epithelial integrity, immune regulation, and antiviral defense [175,176,177,178]. The key pathways through which probiotics and postbiotics may exert therapeutic effects in post-COVID are shown in Figure 4.

##### Tight Junction Modulation and Barrier Function

Tight junction (TJ) proteins, including claudins, occludins, and zonula occludens (ZO), maintain the integrity of the epithelial barrier that prevents the passage of various molecules and microorganisms through intercellular spaces. Certain probiotic strains, such as *Lactiplantibacillus plantarum*, *Latilactobacillus sakei* and *Weissella cibaria*, have been shown to increase TJ protein expression, thereby reducing intercellular permeability and preventing microbial translocation [184]. Enhanced contact between epithelial cells is an important mechanism underlying the antiviral effect of probiotics contributing to intestinal health and overall immunity [185]. Interestingly, water extracts of postbiotics from *Bacillus amyloliquefaciens* J and *L. plantarum* SN4 enhanced the expression of TJ proteins, such as occludin, ZO-1, and claudin, which are essential for maintaining the integrity of the GI barrier. This helped in preventing pathogen invasion (e.g., *E. coli*-induced enteritis) and maintaining GI health [186]. Probiotic supplementation (i.e., *Bacillus licheniformis* S6 or *L. plantarum* ZLP001) has been shown to increase ZO-1 and occludin expression in intestinal epithelial cells [187,188,189]. Butyrate SCFA, a key postbiotic, also supports barrier integrity by promoting mucin secretion, histone acetylation, and anti-inflammatory signaling through inhibition of G protein-coupled receptors (i.e., GPR41/43) and *histone deacetylases* (HDAC) [190].

Notably, some viruses, including adenoviruses and Coxsackie viruses, can use TJ proteins as entry receptors, highlighting the importance of strengthening epithelial barriers in antiviral defense [191].

##### Inhibition of Viral Fusion

Certain probiotic LAB strains can bind directly to viral particles or compete for cell receptors, preventing viral particles from entering cells [179]. Direct interactions are more likely to occur through the adsorption or trapping viral particles by LAB cells [180]. Some strains, such as *Lacticaseibacillus paracasei* A14, *L. paracasei* F19, *L. paracasei/rhamnosus* Q 85, *L. plantarum* M1.1, and *Limosilactobacillus reuteri* DSM 12246, have been shown to capture vesicular stomatitis virus (VSV), possibly by masking viral fusion domains or competing for membrane receptors [192]. These actions affect specific stages of the virus life cycle, such as attachment and entry. In turn, Wang et al. [193] observed that *Enterococcus faecium* strain NCIMB 10415 effectively inhibited swine flu virus through direct interactions.

##### Bioactive Postbiotics and Specific Microenvironment

One of the important processes carried out by probiotics is the production of various metabolic substances. Metabolites (postbiotics) are a variety of biologically active compounds produced as a result of microbial activity. These compounds include H_2_O_2_, lactic acid, SCFAs, and AMPs [179,194], particularly bacteriocins [22]. Research evidence suggests that these metabolites can exert antiviral effects by creating a specific microenvironment that prevents viral replication, thereby significantly reducing their impact on the body. For example, probiotic bacterial strains play a significant role in maintaining the balance of the vaginal microbiota. These beneficial microorganisms, mainly LAB, are actively involved in inhibiting the growth of pathogens by maintaining an optimal pH and producing lactic acid, H_2_O_2_, and other antimicrobial substances. Studies have shown that H_2_O_2_ produced by a probiotic *L. acidophilus* strain has a toxic effect on human immunodeficiency virus (HIV) and can block its replication [181]. Other studies have shown the inhibitory activity of lactic acid, also produced by members of the *Lactobacillus* group, against HIV and herpes simplex virus (HSV) [195,196].

##### Antimicrobial Peptides and Selective Pathogen Targeting

AMPs are a group of low-molecular-weight short-chain peptides with protein-like activity, capable of killing microorganisms, including bacteria, viruses, protozoa, and fungi [179]. AMPs may play a significant role as part of a novel therapeutic strategy in the future. As the number of antibiotic-resistant microorganisms has increased, the use of AMPs may lead to the development of new treatments for various infections. A specific subclass of AMPs synthesized by bacteria is referred to as bacteriocins. They have antimicrobial activity against other bacteria, but bacteria that produce these compounds are resistant to their effects [197]. Over the years, an increasing number of studies have been conducted, indicating the potential of bacteriocins as antiviral agents. One of the first bacteriocins tested for antiviral activity was enterocin CRL35 produced by *E. faecium* CRL35. Wachsman et al. [198] reported that enterocin CRL35 had an inhibitory effect against HSV-1 and HSV-2 strains [182]. Todorov et al. [183] studied the antibacterial and antiviral activity of the ST4V peptide produced by the *Enterococcus mundtii* ST4V strain. They found that, in addition to strong activity against some bacterial strains, the peptide exerted inhibitory activity against some viruses, such as HSV-1, HSV-2, and measles virus (MV, *Measles morbillivirus*), and to a lesser extent against poliomyelitis virus (PV3). These AMPs could interfere with viruses by blocking receptor attachment, disturbing viral envelope integrity, or aggregating viral particles [182,183].

Importantly, AMPs exhibit selective activity due to differences in the cell membrane composition and charge, which allows them to distinguish pathogens from host cells [179]. This selectivity is crucial for maintaining the microbiota balance while reducing the pathogen burden [198].

##### Cytokine and Chemokine Regulation

An equally important strategy employed by probiotic bacterial strains against viral particles is the modulation of the host immune system. Probiotics can modulate host cytokine profiles by interacting with pattern recognition receptors (PRRs), such as Toll-like receptors (TLRs) and NOD-like receptors (NLRs). These interactions lead to differential activation of the NF-κB, interferon regulatory factor 3 (IRF3), and MAPK pathways, resulting in altered expression of IL-10, IL-6, TNF-α, and interferons (IFN-γ) [199,200,201]. In response to the virus, host cells begin to secrete IFN, which helps inhibit viral replication in infected cells. IFN also helps activate other cells in the immune system, which strengthens the antiviral immune response. Some studies have shown that *L. rhamnosus* CRL1505 can induce IFN type I in intestinal antigen-presenting cells (APCs), thereby inhibiting viral infections [202,203]. IL-12 is considered one of the major components of the human immune response. An important function of this cytokine is to stimulate various cells of the immune system to destroy infected cells and inhibit viral replication. According to the observations reported by Kawashima et al. [204], the bacterial strain *L. plantarum* YU isolated from Japanese fermented products intensively stimulated IL-12 production in mouse peritoneal macrophages and increased the activity of natural killer cells (NK-cells).

Briefly, probiotics and postbiotics influence post-COVID-related pathophysiology at the molecular level through barrier stabilization, cytokine modulation, AMP production, and viral interference. These mechanisms offer opportunities for targeted microbiome-based interventions in the treatment of post COVID.

#### 4.2.2. Efficacy of Probiotics Against SARS-CoV-2

Currently, the use of probiotics for the treatment of COVID-19 has attracted the attention of both medical professionals and the public. It is known that the viral disease caused by SARS-CoV-2 manifests differently in each person and can lead to serious complications. While scientists are mainly focused on developing vaccines and using drugs, there are opinions that probiotic bacterial strains can improve the host response to infection. Some studies suggest that carefully selected probiotic preparations may have a positive effect on immune function, which could theoretically help the organism fight the virus [172,173,205].

A decrease in the effectiveness of the body’s immune system is caused by disruption of the normal composition of microorganisms. It should be noted that certain bacterial groups (e.g., *Escherichia*/*Shigella*, *Clostridioides difficile*) that stimulate inflammatory processes can significantly increase the production of pro-inflammatory cytokines, leading to a cytokine storm and contributing to additional tissue and organ damage [206]. Therefore, it is important to focus on more comprehensive exploration of new ways to treat gut microbiota dysbiosis, thereby strengthening the immune system, which will greatly help to alleviate the patient’s condition and succeed in the fight against viral infection. The use of probiotics in patients with COVID-19 (Table 4) as an adjunctive treatment to boost immunity may prove to be a promising method with high efficacy in activating the body’s natural defense mechanisms.

A meta-analysis conducted by Tian et al. [170], who analyzed 10 randomized clinical trials involving 1198 patients with confirmed COVID-19, showed that probiotics increased the number of patients with overall improvement in disease symptoms and shortened the duration of their symptoms and hospitalization, compared with the control group. No clear effect of probiotics was observed on such symptoms as fever, headache, and weakness. In the case of inflammation, probiotics can effectively reduce the concentration of CRP by reducing the inflammatory response in the body. In addition, probiotics have been shown to alleviate GI symptoms (e.g., improving GI microbiota and reducing the duration of diarrhea) and improve respiratory symptoms by reducing the duration of shortness of breath through the gut-lung axis.

However, the quality of these RCTs and meta-analysis varies, and some do not include detailed descriptions of randomization and allocation concealment, which may lead to potential systematic errors [214,215]. The patient populations in these studies include both the general group of COVID-19 patients and specific subgroups, such as severe cases, immunocompromised individuals, and patients with GI symptoms [216,217,218,219]. This heterogeneity can affect the generalizability of the findings. The sample size varies greatly, ranging from small groups to large-scale studies involving hundreds of thousands of participants [217,220,221]. Smaller samples (*n* < 100) may limit the statistical power and reliability of the results. There is considerable diversity in the types of probiotics used, including different strains and doses [218,221,222]. This lack of standardization makes it difficult to compare studies and formulate general recommendations. These studies show a wide range of outcomes, from symptom reduction and shortened illness duration to mortality rates and immune response modulation [214,220,221]. This variability in outcome measures further complicates the synthesis of results.

Considering the above, probiotics could potentially have significant beneficial effects, such as symptom reduction, immune modulation, and reduction in mortality and hospitalization rates among COVID-19 patients receiving probiotics, although these findings are not universally consistent [214]. However, their exact mechanisms of action in combating SARS-CoV-2 are not yet fully understood. Additional research in this area is vital, as it will help identify the most effective probiotic strains, optimal doses, and administration regimens.

## 5. Use of Probiotics to Alleviate Post-Acute COVID-19 Syndrome

Probiotics, prebiotics, and postbiotics show promise as additional post-COVID treatments (Table 5).

Probiotics administered at a dose of over 10 billion CFU (Colony Forming Unit) per day for at least 4 weeks appeared to have a positive effect on alleviating post-COVID, in particular, increasing SCFA-producing bacteria, such as *Faecalibacterium* and *Bifidobacterium.* After the administration of the synbiotic (i.e., SIM01), there was a significant increase in the abundance of probiotic bacteria, such as *B. longum*, *Bifidobacterium pseudocatenulatum*, *Roseburia intestinalis*, *Roseburia hominis*, *F. prausnitzii*, and *Akkermansia muciniphila* with a simultaneous decrease in the abundance of pathogenic bacteria, such as *Ruminococcus gnavus*, *Klebsiella pneumoniae*, *Klebsiella variicola*, and *Parabacteroides merdae* [223]. The increase in the SCFA levels and related microorganism populations correlated with the progress in recovery.

**Figure 5:**
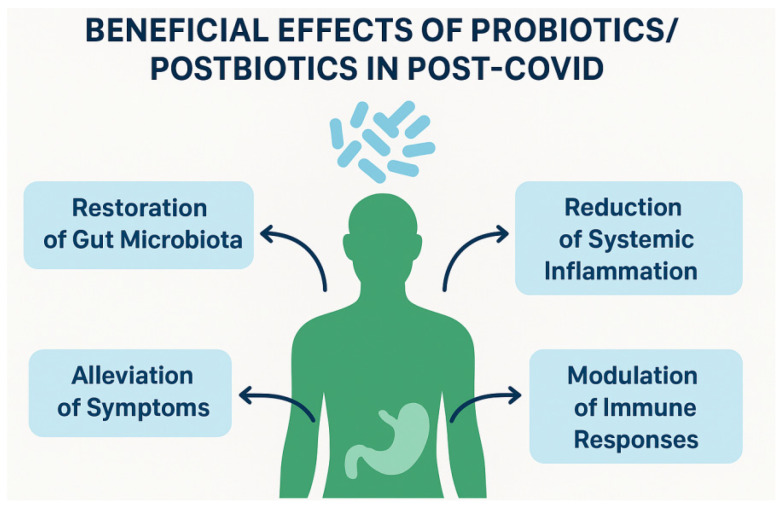
Beneficial effect of probiotics and prebiotics in post-COVID [223,224,225,226,227].

Although several RCTs and meta-analyses indicated beneficial effects of probiotics and postbiotics in post-COVID (Figure 5), the overall evidence base has significant limitations. Many of the clinical trials show considerable methodological heterogeneity, including differences in intervention duration, outcome measures, and follow-up periods. In addition, the sample sizes are often small (frequently *n* < 100 participants), limiting the statistical power to detect clinically relevant effects. The probiotic preparations themselves vary considerably in terms of strains, doses, and methods of administration as well as various additives, such as plant extracts, macroelements, or fibers, making comparisons between studies difficult. Furthermore, most studies did not stratify their results based on key patient variables, such as age, sex, baseline microbiota composition, or comorbidities. Collectively, these factors make it difficult to draw definitive conclusions or formulate generalized clinical recommendations.

### 5.1. Neuropsychological Manifestations of Post-COVID

#### 5.1.1. Effect of Probiotics on Fatigue

Fatigue is one of the most common symptoms in patients with post-COVID [93]. Some sources report that, during the acute phase of SARS-CoV-2 infection, fatigue affected 90% of patients [228]. In some individuals, this symptom persisted for six months after the onset of the disease, progressing to the chronic phase [229]. Even a year after the initial infection, increased fatigue was reported in 40% of individuals [102]. Patients complained of severe fatigue that did not subside even after prolonged sleep or rest. Many individuals had difficulty performing normal daily activities, which significantly reduced their quality of life. It has been reported that fatigue symptoms in patients can worsen over time [229]. A significant correlation was found between previously diagnosed depression and antidepressant use and the later onset of severe fatigue [102]. In a systematic review, Joli et al. [230] found an increased risk of experiencing fatigue after SARS-CoV-2 infection in elderly patients and women. A longer acute phase of the disease, as well as a more severe course of the disease, has also been shown to increase the risk of fatigue. Some patients often report brain (mental) fog when they talk about fatigue and indicate that these symptoms worsen throughout the day [231]. Those experiencing the brain fog often have difficulty concentrating, memory problems, and reduced information processing speed [232,233]. Patients often complain of difficulty performing activities that require mental effort and attention. These symptoms have a strong negative impact on various aspects of patients’ lives, affecting interpersonal relationships, work, and daily activities.

Caprioli et al. [225] conducted a randomized clinical trial with a placebo to determine the efficacy of the probiotic supplement VSL#3^®^ in patients with post-COVID, with a particular focus on fatigue. The formula contained a mixture of various live lyophilized strains of probiotic bacteria, i.e., *S. thermophilus* BT01, *B. breve* BB02, *B. lactis* BL03, *B. lactis* BI04, *L. acidophilus* BA05, *L. plantarum* BP06, *L. paracasei* BP07, and *L. helveticus* BD08. The analysis involved 38 participants, 19 of whom received a probiotic supplement and the other half took a placebo for 4 weeks. Fatigue in patients was assessed using the Chalder Fatigue Scale (CFS). The CFS consists of 11 items, each rated on a four-point scale. The questions covered two main areas, i.e., physical and mental fatigue. After the treatment, a significant decrease in the Chalder’s fatigue index was observed in the group taking the probiotics. In contrast, patients receiving the placebo showed no change in the level of perceived fatigue.

ProbioSEB CSC3 has also proven effective against fatigue in combination with ImmunoSEB’s systemic enzymes (enterosolubilized serrapeptase, bromelain, amylase, lysozyme, peptidase, catalase, papain, glucoamylase, and lactoferrin). It contains several bacterial strains, including *Bacillus coagulans* LBSC (DSM 17654), *Bacillus subtilis* PLSSC (ATCC SD 7280), and *Bacillus clausii* 088AE (MCC 0538). After two weeks of taking these supplements, fatigue symptoms disappeared in 91% of the study participants. The participants in the experimental group showed a significant reduction in physical and mental fatigue at all stages of the study, compared with the control group [234].

Interestingly, the administration of probiotics as early as at the onset of COVID-19 has a significant effect on later perception of fatigue in patients. A study conducted by Santinelli et al. [226] showed that administration of a multi-strain supplement, Sivomixx800R, containing probiotic bacteria *S. thermophilus* DSM 32245 R, *B. lactis* DSM 32246 R, *B. lactis* DSM 32247 R, *L. acidophilus* DSM 32241 R, *L. helveticus* DSM 32242 R, *L. paracasei* DSM 32243 R, *L. plantarum* DSM 32244 R, and *Lactobacillus brevis* DSM 27961 R at a dose of 2.4 billion CFU 3 times daily throughout hospitalization of COVID-19 patients may prevent the development of chronic fatigue by affecting key metabolites involved in glucose utilization and energy pathways. The supplementation with this probiotic had a positive effect on metabolic homeostasis by negatively affecting the occurrence of CNS-related symptoms after hospital discharge. Of the 58 hospitalized COVID-19 patients, 24 received Sivomixx800R during hospitalization, whereas 34 received only standard treatment. Six months after discharge from the hospital, the participants’ feelings were assessed using the CFS. 70.7% of participants reported fatigue. However, the group taking the probiotic showed a significantly lower percentage of fatigue reports than the control group. In addition, the patients in the probiotic group had significantly increased serum levels of arginine, aspartate, and lactate and lower levels of 3-hydroxyisobutylate than those not treated with probiotics. These results suggest that probiotic administration in COVID-19 may have long-term health benefits for recovering patients, as the presence of post-COVID suggests chronic CNS involvement in COVID-19.

#### 5.1.2. Effect of Probiotics on Depression

A high prevalence of neuropsychological symptoms, such as depression, anxiety, and cognitive impairment, has been observed in patients after SARS-CoV-2 infection. Approximately 40% of patients experience clinically significant depressive symptoms 1, 3, 6, and 12 months after COVID-19 [235]. In their meta-analysis, Deng et al. [236] highlighted that hospitalized patients exhibited a higher prevalence of depressive symptoms compared to outpatients, at 48% and 35%, respectively. Some studies have shown that subjects with a more severe course of infection are at a higher risk of developing depression than those with clinically stable COVID-19. Several risk factors contributing to this condition in patients who have survived SARS-CoV-2 infection have been described, including poor mental stability, enforced isolation after recovery, lack of social support, and physical symptoms (e.g., poor sleep quality, fatigue, or palpitations) [237]. In addition, searching the media for data on the spread of the virus has been shown to have a negative impact on depressive symptoms [235].

There is growing evidence of a link between episodes of depression and changes in the patient’s gut microbiota. It has been suggested that imbalances in the gut microbiota may modulate the inflammatory response in individuals with depression by attenuating the effect of pro-inflammatory cytokines. This may directly induce the development of depression [238]. There is some evidence for this mechanism, reporting that people experiencing depression have elevated levels of pro-inflammatory cytokines, such as IL-1β and IL-6, and reduced levels of anti-inflammatory cytokines, such as IL-4 and IL-10 [239]. Gut microbiota can also influence neurotransmitter levels in the body by producing metabolites that can affect mental health. Some studies have shown that SCFAs increase serotonin production in the gut [240].

Wallace and Milev [241] demonstrated the positive effects of the probiotic supplement CEREBIOME^®^ containing *Lactobacillus helveticus* R0052 and *Bifidobacterium longum* R0175 on alleviating depressive symptoms in patients with moderate clinical depression. The participants showed positive dynamics in improved mood, reduced anxiety, and improved sleep quality. In their study, Ghorbani et al. [242] examined the effects of the synbiotic Familact H^®^ containing strains of *Lacticaseibacillus casei*, *L. acidophilus*, *Lactobacillus bulgaricus*, *L. rhamnosus*, *B. breve*, *B. longum*, *S. thermophilus*, and fructooligosaccharides as a prebiotic. A group of 20 patients with depressive disorders received the synbiotic along with fluoxetine, whereas the other participants received fluoxetine and a placebo. As a result, patients taking the synbiotic as an adjunct therapy to fluoxetine had significantly lower levels of depression on the Hamilton Rating Scale for Depression (HAM-D), compared to the control group. In addition, a comprehensive review found that the use of probiotics and a high-fiber diet, especially in combination with antidepressants, in patients with depression can have a positive impact on their recovery [66]. Moreover, increasing dietary fiber (prebiotics) intake can change the gut microbiota composition, which may reduce digestive and non-digestive symptoms, such as anxiety and palpitations in individuals with post-COVID [243].

### 5.2. Gastrointestinal Symptoms of Post-COVID

#### Effect of Probiotics on Irritable Bowel Syndrome (IBS)

IBS is a GI disorder characterized by abdominal pain, bloating, and changes in stool structure [244]. Patients infected with SARS-CoV-2 may also experience this syndrome. The exact mechanisms underlying IBS are not yet fully understood, but it has been suggested that such factors as impaired intestinal motility, chronic inflammation, changes in the GI microbiota, and psychological stress may influence the development of this syndrome [245]. In a systematic review conducted by Dale et al. [246], seven of 11 studies confirmed the positive effects of probiotic supplementation in patients with IBS compared to a placebo group. Majeed et al. [247] tested the efficacy of probiotic *B. coagulans* strain MTCC 5856 along with standard treatment (domperidone 30 mg + ezomeprazole 40 mg and metronidazole 400 mg) in patients with IBS for 90 days. The study group showed improvement in such symptoms as abdominal bloating, vomiting, diarrhea, and stool frequency compared to the placebo group. In contrast, Mezzasalma et al. [248] conducted a study involving three groups of IBS patients with predominant constipation, with two groups taking probiotic preparations and one receiving a placebo. The first group received a probiotic containing strains of *L. acidophilus* and *L. reuteri*, and the second group received *L. plantarum*, *L. rhamnosus*, and *B. lactis* for 60 days. Before the analysis, patient complaints included cramping, bloating, abdominal pain, and constipation. It was found that both groups taking the probiotics significantly improved in all the aforementioned symptoms, in contrast to the group taking the placebo. Similarly, a study conducted by Krammer et al. [249] demonstrated the effectiveness of *L. plantarum* strain 299v in 221 patients with IBS who received the supplement for 12 weeks. The probiotics significantly relieved all the IBS symptoms. A reduction in abdominal pain episodes, a decrease in bloating, and normalization of stool frequency were achieved.

To date, very few studies have confirmed the efficacy of probiotics in the treatment of COVID-19-related IBS. However, the overall beneficial effects of probiotics on GI health and relief of non-COVID-19-related IBS symptoms have been confirmed in numerous studies. Therefore, the use of already available treatments for IBS has the potential to improve patient’s health and significantly improve their quality of life.

## 6. Conclusions

Current literature confirms the important role of the GI microbiota and its modulation in the context of post-COVID. Probiotics and postbiotics offer promising opportunities to alleviate inflammation, restore mucosal barrier integrity, and support gut–brain and gut-immune axis function. Although many clinical studies suggest improvement in symptoms, particularly fatigue, GI complaints, and cognitive impairment, the heterogeneity of strains, doses, study designs, and patient populations remains a challenge.

Current evidence suggests that such strains as *L. rhamnosus*, *L. plantarum*, and *B. longum*, alone or in combination with dietary fiber or synbiotics, may be effective in alleviating post-COVID symptoms. Doses typically range from 1 to 10 billion CFU/day, depending on the preparation. In addition, postbiotic compounds, such as SCFAs and bacteriocins, have demonstrated molecular activity relevant to both antiviral and immunomodulatory responses.

Therefore, although further research is needed, particularly high-quality standardized RCTs, the therapeutic modulation of the GI microbiota may become a valuable component of post-COVID treatment strategies. Post-COVID is a complex condition that requires close medical supervision. It is important to remember that each patient has different symptoms and may present differently; therefore, a personalized approach to treating and managing these symptoms is crucial for successful recovery from COVID-19 infection.

### Future Direction

Future research should aim to

- Identify optimal probiotic and postbiotic formulations based on specific post-COVID phenotypes;
- Standardize clinical trial endpoints and stratify outcomes according to age, comorbidities, baseline microbiota profiles, and COVID-19 severity;
- Evaluate synbiotic approaches combining probiotics with targeted prebiotics or bioactive compounds;
- Examine the regulatory environment and safety profiles of novel postbiotic therapies.
- Employ omics technologies (metabolomics, proteomics, transcriptomics) to better understand host-microbiota interactions;
- Investigate long-term outcomes and sustainability of microbiota changes after recovery from COVID-19;
- Incorporate microbiome-based interventions into broader rehabilitation programs that include elements of nutritional, psychological, and physical therapy;
- Recent findings suggest a potential role for bacteriophages in modulating the composition of the gut microbiota and the host immune response in the context of post-COVID conditions [250]. However, this is still a new field that requires further mechanistic and clinical research. Bacteriophages are being studied as a separate, microbiota-targeting strategy, which may complement or serve as a parallel method to probiotic/postbiotic interventions.

These directions will help develop more personalized and evidence-based microbiota-targeted therapies for post-COVID.

## Figures and Tables

**Table 1:** Prevalence of the most commonly experienced symptoms of post-COVID [99,100,101,102].

Symptoms of Post-COVID	Prevalence (%)
Fatigue	40–58
Headache	44
Concentration disturbances (attention deficits)	27
Hair loss	25
Dyspnea	24
Gastrointestinal disorders	22
Loss of appetite	20
Irritable bowel syndrome (IBS)	17–39
Loss of taste	17
Abdominal pain	14

**Table 2:** Post-COVID patient-specific factors affecting health after COVID-19 conditions [104,105,106,107,108,109,110,111,112,113,114,115,116,117].

Post-COVID Patient-Specific Factors	Impact on Post-COVID Conditions
Older age	Higher risk of severe outcomes and post-COVID symptoms, such as fatigue and cognitive impairments
Younger age	Significant mental health issues, such as anxiety and depression
Female sex	Higher levels of persistent post-COVID symptoms, such as anxiety, depression, and post-traumatic stress disorder
Diabetes	Increased risk of severe outcomes and persistent symptoms
Hypertension	Higher incidence of post-COVID
Obesity	Worse post-COVID outcomes and prolonged symptoms
Mental health	Exacerbation of post-COVID symptoms, higher rates of depression and anxiety
Other comorbidities	Chronic conditions, such as cardiovascular and kidney diseases, contribute to severe outcomes
Inflammatory markers	Elevated IL-6 levels linked to post-COVID
Microbiota profiles	Potential influence on immune response and inflammation
Genetic factors	Significant influence on post-COVID health outcomes, affecting both the severity of the initial infection and long-term consequences

**Table 3:** Basic functions and mechanisms of action of probiotics [160].

Functions of Probiotics	Mechanism of Action of Probiotics
Restoration of normal intestinal microbiota	Restoration of a healthy intestinal microbiota by colonizing the epithelium and competing with pathogens thus ensuring microbial balance.
Production of antimicrobial agents	Production of bacteriocins and organic acids inhibiting the growth and reproduction of pathogens.
Modulation of enzymatic activity and toxin metabolism	Participation in the metabolism of toxic substances and production of volatile fatty acids contributing to energy balance.
Increased intestinal cell adhesion and mucin production	Strengthening the intestinal barrier by increasing cell adhesion and producing a protective layer of mucus (mucins).
Regulation of the activity of the immune system and lymphoid tissue	Participation in regulating the immune system and maintaining healthy intestinal lymphatic tissue.

**Table 4:** Selected randomized clinical trials on the use of probiotics in the treatment of COVID-19.

Strains Used	Daily Dose in CFU	Number of Patients (*n*)	Test Results	Reference
*Streptococcus thermophilus* DSM 32345; *Lactobacillus acidophilus* DSM 32241; *Lactobacillus helveticus* DSM 32242; *Lacticaseibacillus paracasei* DSM 32243; *Lactiplantibacillus plantarum* DSM 32244; *Lactobacillus brevis* DSM 27961; *Bifidobacterium animalis* subsp. *lactis* DSM 32246; *Bifidobacterium lactis* DSM 32247	2.4 × 10^12^ CFU for 7 days	*n* = 70: probiotic *n* = 28; placebo *n* = 42	Reduction in diarrhea, shortness of breath, cough, and fever, and the risk of respiratory failure was 8 times lower in patients taking the probiotic	[207]
*Bifidobacterium animalis* subsp. *Lactis* BB-12	1 × 10^12^ CFU for 3 days	*n* = 44: probiotic *n* = 20; without probiotic *n* = 24	In 95% of patients who received the probiotic, the average hospital stay was reduced by an average of 7.6 days compared to the control group (an average of 13.6 days); the decrease in the mortality rate was 5% in the probiotic group versus 20.83% in the non-probiotic group.	[208]
*Lactiplantibacillus plantarum* KABP022, *Lactiplantibacillus plantarum* KABP023, *Lactiplantibacillus plantarum* KAPB033; *Pediococcus acidilactici* KABP021	2 × 10^9^ CFU for 30 days	*n* = 293: probiotic *n* = 147; placebo *n* = 146	53.1% of patients taking the probiotic experienced remission (elimination of symptoms and virus); 28.1% of patients in the placebo group experienced remission. Patients taking the probiotic experienced a reduction in the number of days with fever, headache, cough, shortness of breath, and body aches	[209]
*Streptococcus salivarius* K12	1 × 10^9^ CFU for 14 days	*n* = 50: probiotic *n* = 25; placebo *n* = 25	Probiotic administration contributed to colonization of the oral environment, improved blood parameters, and reduced mortality in patients with COVID-19 from 32% in the non-probiotic group to 8% in the probiotic group	[210]
*Limosilactobacillus reuteri* DSM 17938 Vitamin D3	2 × 10^8^ CFU 20 μg for 6 months	*n* = 132: Probiotic with D3 *n* = 70, including *n* = 15 vaccinated; Placebo with D3 *n* = 62, including *n* = 15 vaccinated;	Long-term enhancement of the immune response, especially in those fully vaccinated with the mRNA vaccine (at 28 days post-vaccination), who showed higher levels of anti-RBD IgA	[211]
*Lacticaseibacillus rhamnosus* GG (LGG) as post-exposure prophylaxis for COVID-19	10 × 10^9^ CFU for 28 days	*n* = 182: LGG *n* = 91; placebo *n* = 91	LGG was associated with a prolonged time to COVID-19 infection and reduced the incidence of illness symptoms and gut microbiome changes when used as prophylaxis for more than 7 days post-COVID-19 exposure, but not the overall incidence	[212]
*Lactobacillus (L.) rhamnosus*, *L. helveticus*, *L. casei*, *Bifidobacterium (B.) lactis*, *L. acidophilus*, *B. breve*, *L. bulgaricus*, *B. longum*, *L. plantarum*, *B. bifidum*, *L. gasseri*, and *Streptococcus (S.) thermophilus*, fructooligosaccharides-prebiotic agent	2 × 10^9^ CFU for 2 weeks	*n* = 78: probiotic *n* = 39; placebo *n* = 39	A significant reduction in pro-inflammatory markers like IL-6 and improvements in white blood cell counts in hospitalized COVID-19 patients; other findings showed no statistical differences between groups	[213]

Explanations: CFU—colony forming unit; IgA—immunoglobulin class A; RBD—receptor binding protein.

**Table 5:** Probiotics and postbiotic metabolites in post-COVID symptom management in randomized placebo-controlled trials.

Probiotic Strain(s)	Daily Dose/Metabolites/Postbiotic Compounds	Number of Patients (*n*)	Targeted Symptoms	Outcomes	Reference
Synbiotic blend SIM01: *Bifidobacterium adolescentis*, *Bifidobacterium longum*, *Bifidobacterium bifidum*, prebiotics such as galacto-oligosaccharides, xylooligosaccharides, resistant dextrin	10 × 10^9^ CFU in sachets twice daily for 30 days/SCFAs, tryptophan metabolites	*n* = 463: SIM01: *n* = 232 Placebo: *n* = 231	Fatigue, memory loss, hair loss, GI symptoms,	Significant improvement in fatigue, memory, and GI symptoms; significant increase in SCFA-producing strains	[223]
Synbiotic blend (DSM-Firmenich): *Lacticaseibacillus rhamnosus* DSM 32550, *Lactiplantibacillus plantarum* DSM 34532 (Humiome^®^), *Bifdobacterium lactis* DSM 32269, *Bifdobacterium longum* DSM 32946, 2.5 g of prebiotic fiber fructooligosaccharides, 5 mg of zinc	The dosage of probiotics was not specified, for 3 months/ creatine, SCFAs	*n* = 26: probiotics *n* = 13; placebo *n* = 13	Post-exertional malaise, cognitive function	Improved post-exercise recovery and increased brain creatine levels	[224]
VSL#3^®^, a combination of 3 *Bifidobacterium* strains (*B. breve*, *B. longum*, *B. infantis*), 4 strains of *Lactobacillus* s.l. (*L. acidophilus*, *L. plantarum*, *L. casei*, *L. bulgaricus*), *Streptococcus thermophilus*	450 × 10^9^ CFU in sachets twice daily for 28 days/no measurement of any probiotic-derived metabolites or postbiotics	*n* = 38: probiotics *n* = 19; placebo *n* = 19	Fatigue, physical functioning, GI symptoms	Significant decrease in fatigue, significant amelioration in physical functioning, and improvement in GI symptoms; VSL#3^®^ treatment was not associated with improvements in symptoms of anxiety, depression, performance, or somatization	[225]
SLAB51 (Sivomixx800^®^)-a probiotic mixture: 5 strains of *Lactobacilli* s.l. (*L. acidophilus*, *L. helveticus*, *L. paracasei*, *L. plantarum*, *L. brevis*), 2 strains of *Bifidobacterium* (*B. lactis* DSM 32246^®^, *B. lactis* DSM 32247^®^), *Streptococcus thermophilus*	2400 × 10^9^ CFU daily for 6 months/arginine, asparagine, lactate, and 3-hydroxyisobutyrate	*n* = 58: probiotics + antibiotics *n* = 24; placebo + antibiotics *n* = 34	Fatigue	Decrease in chronic fatigue; higher levels of serum lactate, asparagine, and arginine in the probiotic group; 3-Hydroxyisobutyrate levels were significantly lower in the probiotic-treated participants compared to controls	[226]
Synbiotic capsule: 5 strains of *Lactobacillus* s.l. (*L. plantarum*, *L. rhamnosus*, *L. bulgaricus*, *L. lactis* and *L. paracasei*) +200 mg of prebiotic Inulin daily; phytochemical rich capsule (PC): *Citrus Sinensis* fruit (400 mg from 200 mg of 2:1 extract, standardized to contain 70 mg of Bioflavonoids); Chamomile, *Matricaria recutita* L. flower) (1000 mg from 22 mg of 10:1 extract and 65 mg of 12:1 extract). *Curcuma longa* rhizome in Curcumin Complex (1600 mg of curcumin from 25 mg of 64:1 extract, standardised to contain 23.8 mg of curcuminoid). Pomegranate (*Punica granatum* L. rinds and seeds) (1000 mg from 25 mg of 40:1 extract, standardised to contain 10 mg of Ellagic Acid). *Polygonum cuspidatum* root containing 100 mg of resveratrol	10 × 10^9^ CFU daily for 30 days/no measurement of any probiotic-derived metabolites or postbiotics, herb substances	*n* = 147: synbiotic + PC *n* = 74 Placebo *n* = 73	Fatigue, physical functioning, common toxicity symptoms, cough scores, subjective well-being scores, duration of pyrexia	Decrease in cough score and decrease in the fatigue score in the synbiotic group; improvement in the overall well-being score; mild bloating in two participants.	[227]

Explanations: CFU—Colony Forming Unit; GI—gastrointestinal; SCFAs—Short-chain fatty acids.

## Data Availability

No new data were created or analyzed in this study. Data sharing is not applicable to this article.

## Abbreviations

AAD	Antibiotic-associated diarrhea
ACE2	Angiotensin-converting enzyme 2
AMPs	Antimicrobial peptides
APCs	Antigen-presenting cells
CD	Crohn’s disease
CFS	Chalder Fatigue Scale
CFU	Colony forming unit
CNS	Central nervous system
FAO	Food and Agriculture Organization
GI	Gastrointestinal
GPR	G protein-coupled receptors
HAM-D	Hamilton Rating Scale for Depression
HDAC	*Histone deacetylases*
HIV	Human immunodeficiency virus
HPA	Hypothalamic–pituitary–adrenal
HSV	Human simplex virus
IBD	Inflammatory Bowel Disease
IBS	Irritable bowel syndrome
IFN	Interferon
IgA	Immunoglobulin class A
IL	Interleukin
IRF	Interferon regulatory factor
LAB	Lactic acid bacteria
LPS	Lipopolysaccharides
MV	Measles virus
NF-κB	Nuclear factor kappa B
NK	Natural killer
NLRs	NOD-like receptors
PACS	Post-acute COVID-19 syndrome
PCS	Post-COVID-19 syndrome
PGF	Placental growth factor
POTS	Postural orthostatic tachycardia syndrome
PRRs	Pattern recognition receptors
PV	Poliomyelitis virus
RBD	Receptor binding protein
S	Spike
s.l.	Sensu lato
SARS-CoV-2	Severe Acute Respiratory Syndrome Coronavirus 2
SCAFs	Short-chain fatty acids
SIP	sphingosine-1-phosphate
TJ	Tight junction
TLRs	Toll-like receptors
UC	Ulcerative colitis
VOCs	Variants of concern
VSV	Vesicular stomatitis virus
VUM	Variant under monitoring
WHO	World Health Organization
ZO	Zonula occludens
